# Assessing equivalent and inverse change in genes between diverse experiments

**DOI:** 10.3389/fbinf.2022.893032

**Published:** 2022-09-21

**Authors:** Lisa Neums, Devin C. Koestler, Qing Xia, Jinxiang Hu, Shachi Patel, Shelby Bell-Glenn, Dong Pei, Bo Zhang, Samuel Boyd, Prabhakar Chalise, Jeffrey A. Thompson

**Affiliations:** ^1^ Department of Biostatistics and Data Science, University of Kansas Medical Center, Kansas City, KS, United States; ^2^ University of Kansas Cancer Center, Kansas City, KS, United States

**Keywords:** differential gene expression, equivalence test, study comparison, diverse experiments, molecular marker

## Abstract

**Background:** It is important to identify when two exposures impact a molecular marker (e.g., a gene’s expression) in similar ways, for example, to learn that a new drug has a similar effect to an existing drug. Currently, statistically robust approaches for making comparisons of equivalence of effect sizes obtained from two independently run treatment vs. control comparisons have not been developed.

**Results:** Here, we propose two approaches for evaluating the question of equivalence between effect sizes of two independent studies: a bootstrap test of the Equivalent Change Index (ECI), which we previously developed, and performing Two One-Sided t-Tests (TOST) on the difference in log-fold changes directly. The ECI of a gene is computed by taking the ratio of the effect size estimates obtained from the two different studies, weighted by the maximum of the two *p*-values and giving it a sign indicating if the effects are in the same or opposite directions, whereas TOST is a test of whether the difference in log-fold changes lies outside a region of equivalence. We used a series of simulation studies to compare the two tests on the basis of sensitivity, specificity, balanced accuracy, and F1-score. We found that TOST is not efficient for identifying equivalently changed gene expression values (F1-score = 0) because it is too conservative, while the ECI bootstrap test shows good performance (F1-score = 0.95). Furthermore, applying the ECI bootstrap test and TOST to publicly available microarray expression data from pancreatic cancer showed that, while TOST was not able to identify any equivalently or inversely changed genes, the ECI bootstrap test identified genes associated with pancreatic cancer. Additionally, when investigating publicly available RNAseq data of smoking vs. vaping, no equivalently changed genes were identified by TOST, but ECI bootstrap test identified genes associated with smoking.

**Conclusion:** A bootstrap test of the ECI is a promising new statistical approach for determining if two diverse studies show similarity in the differential expression of genes and can help to identify genes which are similarly influenced by a specific treatment or exposure. The R package for the ECI bootstrap test is available at https://github.com/Hecate08/ECIbootstrap.

## 1 Introduction

In whole genome differential gene expression studies, the difference in the expression of thousands of genes among groups (e.g., treatment group vs. control group) is investigated with the motivation of finding underlying mechanisms of different conditions such as cancer or to observe treatment effects (Gilbert, 2000). An emerging use for gene expression data is to identify genes that are affected in similar or opposing ways across different studies. There are various reasons for doing so, including validating the results of a study (Fu et al., 2021), finding common underlying mechanisms of a disease (Shen et al., 2016; Blake et al., 2020), or investigating similar treatment effects of different drugs (Hollenbach et al., 2010). One of the important challenges in comparing studies is variable study conditions such as technology, environment, and personnel (Goh et al., 2017). For these reasons, gene expression levels are challenging to directly compare across studies. In many cases though, the focus is on the nature of the change in gene expression between treatment or disease conditions (Gilbert, 2000). Therefore, there is a need for statistical methods that can be used to validate the similarity in expression changes across studies.

Given the lack of statistical methods that can test such equivalence hypotheses, researchers currently use naïve methods for determining if treatments have similar effects on gene expression. The most-commonly employed method focused on intersection, which simply involves finding the intersection of differentially expressed genes across studies, without determining the probability of such intersections occurring by chance. One example is the e-cigarette study from Shen et al. (2016), which investigated if similar pathways were enriched in cigarette smokers. In the process of analyzing their results, they observed that the same or different genes were differentially expressed at several time points of their study and used this result as well as a subsequent gene enrichment study to form their conclusion. Another example is a study from Blake et al. (2020), which compares gene expression across tissues in different animal species. Again, they define genes of interest as being both equivalently changed in the same direction and statistically significant in a pairwise comparison of tissue types. However, because this naïve approach is not statistically motivated, it is prone to false positives, i.e., declaring a gene equivalently changed when the differential expression of the gene between two studies is in the same direction only by chance. This could lead to a large number of candidate genes requiring validation, which could be time-consuming and cost prohibitive (Provenzano and Mocellin, 2007; Garrido et al., 2020).

In this study, we introduce and compare two statistical tests of equivalent change in gene expression between two studies. An advantage to testing for an equivalent change in differential gene expression is that the log2-fold changes are likely to be far more comparable across studies than the gene expression itself due to variable study conditions such as technology, environment, and personnel (Rudy and Valafar, 2011; Walsh et al., 2015). It should be noted that these tests work equally well to find significant opposing changes in gene expression which, for example, could be used to suggest treatments that might reverse changes associated with a disease or to identify genes affected in opposite ways in a gene knockout and overexpression experiment. The first test is an adaptation of the two one-sided t-tests (TOST) applied to the fold-changes from a differential gene expression analysis. Although traditionally the TOST approach has been used to establish bioequivalence of drugs (e.g., to approve generics) (Schuirmann, 1987), it has been adapted to several other cases (Dixon et al., 2018; Leichsenring et al., 2018; Wu et al., 2018), including finding equivalently expressed genes in the same study (Qiu and Cui, 2010). Nevertheless, it has never been adapted to differential gene expression analysis. The second test is based on the Equivalent Change Index (ECI), introduced by Thompson and Koestler (2020) in connection with gene enrichment analysis. Although they introduced the statistic itself, no statistical test was available. Here, we present a bootstrap procedure for the ECI statistic to calculate confidence intervals. In the following, we will explain the mechanisms of both approaches followed by a simulation study to compare and assess the performance of the two methods. This is followed by real-world data applications using different publicly available gene expression studies of pancreatic cancer, Alzheimer’s disease, and smoking vs. vaping to demonstrate if the tests can identify biologically plausible results.

## 2 Methods

In the following, we give a definition of the Equivalent Change Index (ECI), the ECI bootstrap test, and the Two One-Sided t-Tests (TOST). Furthermore, we explain the design of the simulation study and give information about the publicly available data sets used in our real data analysis. For the purpose of this study, we need an effect size ${\hat{\beta }}_{\text{i}}$ , the standard deviation (for TOST) of ${\hat{\beta }}_{\text{i}}$ , and a measurement of statistical significance for ${\hat{\beta }}_{\text{i}}$ (for the ECI bootstrap test) for each differentially expressed gene i . We decided to use the log2 fold change (log2 FC) as effect size, although other measures could also be used, for example the standardized mean difference.

### 2.1 Equivalent change index

The Equivalent Change Index (ECI), proposed by Thompson and Koestler (2020), is a measure of the degree of equivalent or inverse change of attributes of the same type across two diverse studies. The ECI ${\text{λ}}_{\text{i}}$ of a gene i is calculated as a ratio of the minimum and maximum of the absolute effect sizes ${\hat{\beta }}_{\text{ik}}$ from the two studies (k = [1,2]) multiplied by a sign, where the sign reflects whether the differential gene expression of the two studies was in the same direction (positive sign) or opposite direction (negative sign). Furthermore, the ECI is weighted by the maximum of the *p*-values ${\text{p}}_{\text{ik}}$ of the two effect sizes.

$${\text{λ}}_{\text{i}}=\frac{\text{sgn}\left({\hat{\beta }}_{\text{i}1}\times {\hat{\beta }}_{\text{i}2}\right)\text{min}\left(\left|{\hat{\beta }}_{\text{i}1}\right|,\left|{\hat{\beta }}_{\text{i}2}\right|\right)}{\text{max}\left(\left|{\hat{\beta }}_{\text{i}1}\right|,\left|{\hat{\beta }}_{\text{i}2}\right|\right)}\cdot \left(1-\text{max}\left({\text{p}}_{\text{i}1},{\text{p}}_{\text{i}2}\right)\right)$$

${\text{λ}}_{\text{i}}$ is in the range of [-1,1], where -1 indicates that the effect size was exactly opposite between the two studies and 1 indicates that the effect sizes between the two studies were identical. Hence, ${\text{λ}}_{\text{i}}$ indicates the degree of equivalence or inverseness of the expression of a gene compared between two separate experiments.

### 2.2 ECI bootstrap test

**FIGURE 1:**
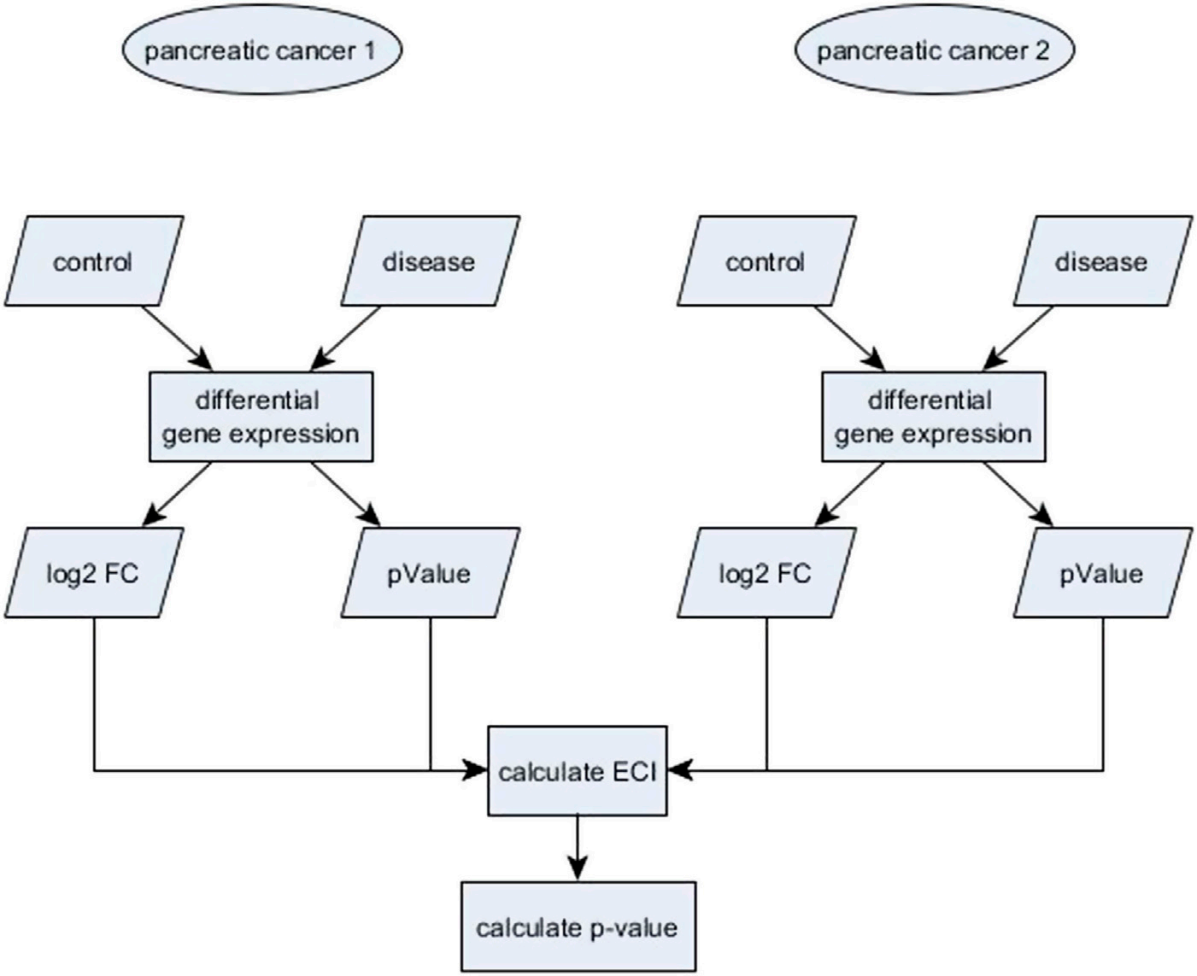
Workflow to compare the differential gene expression of genes of two different studies using the Equivalent Change Index (ECI).

In this section we describe our proposed bootstrap procedure to use the ECI statistic to test for equivalence. We performed all computations in the R environment (R Project for Statistical Computing, RRID:SCR_001905) version 3.5. We first calculate the ECI values for all genes using the function getECI() from the R package ECEA (Thompson and Koestler, 2020) with the log2 FC as effect size and corresponding *p*-values as measurement of statistical significance (Figure 1). To obtain a test measure for equivalent change, we proceed as follows:

• create bootstrap samples within treatment and control groups for each study
• recalculate the differential gene expression for each study separately, e.g., the log2 FC for each gene along with its p-value
• recalculate ECI values between studies for each gene

We repeated this procedure 1,000 times.

#### 2.2.1 Confidence interval

We used a 95% bias-corrected and accelerated (BCa) bootstrap interval as a confidence interval for the ECI values since it corrects for both bias and skewness (Wicklin, 2017; Jung et al., 2019). The BCa assumes that the data are independent and identically distributed and is calculated as $\text{CI}\left[{\hat{\beta }}_{\text{L}}^{\text{*}},{\hat{\beta }}_{\text{U}}^{\text{*}}\right]$ , where ${\hat{\beta }}_{\text{L}}^{\text{*}}$ denotes the *L*th quantile and ${\hat{\text{β}}}_{\text{U}}^{*}$ denotes the *U*th quantile, i.e., lower limit and upper limit. The indices L and U are defined as $\text{L}={\text{a}}_{1}\cdot \text{B}$ and $\text{U}={\text{a}}_{2}\cdot \text{B}$ , where B is the number of bootstrap samples, here B=1000 , and ${\text{a}}_{1}$ and ${\text{a}}_{2}$ are defined as:

$${\text{a}}_{1}\;=\;\Phi \left({\hat{\text{z}}}_{0}+\frac{{\hat{\text{z}}}_{0}+{\text{z}}^{\left(\text{α}/2\right)}}{1\;-\;\hat{\text{a}}\left({\hat{\text{z}}}_{0}+{\text{z}}^{\left(\text{α}/2\right)}\right)}\right)$$

$${\text{a}}_{2}\;=\;\Phi \left({\hat{\text{z}}}_{0}+\frac{{\hat{\text{z}}}_{0}+{\text{z}}^{\left(1-\text{α}/2\right)}}{1\;-\;\hat{\text{a}}\left({\hat{\text{z}}}_{0}+{\text{z}}^{\left(1-\text{α}/2\right)}\right)}\right)$$

Here, Φ is the standard normal cumulative distribution function, ${\text{z}}^{\left(\text{α}/2\right)}$ is the $100\text{x}\left(\frac{\text{α}}{2}\right)$ th percentile of a standard normal distribution, and ${\hat{\text{z}}}_{0}$ and $\hat{\text{a}}$ are the bias-corrections and acceleration factors, respectively. The bias-correction factor is computed as the inverse of the standard normal cumulative distribution function of the proportion of bootstrap effect sizes ${\hat{\text{β}}}^{*}$ smaller than the original effect size $\hat{\text{β}}$ :

$${\hat{z}}_{0}=\Phi^{-1}\left(\frac{1}{B}\overset{B}{\underset{b=1}{\sum }}I_{\left[{\hat{\text{β}}}^{*}<\hat{\text{β}}\right]}\right)$$

The acceleration factor uses “leave one out” (jackknife) resampling as follows:

$$\hat{\text{a}}=\frac{\sum_{\text{i}=1}^{\text{n}}{\left({\hat{\text{β}}}_{\left(.\right)}-{\hat{\text{β}}}_{\left(-\text{i}\right)}\right)}^{3}}{6{\left[\sum_{\text{i}=1}^{\text{n}}{\left({\hat{\text{β}}}_{\left(.\right)}-{\hat{\text{β}}}_{\left(-\text{i}\right)}\right)}^{2}\right]}^{3/2}}$$

Where, ${\hat{\text{β}}}_{\left(-\text{i}\right)}$ is an effect size of a jackknife sample and ${\hat{\text{β}}}_{\left(.\right)}$ is the average of the effect sizes of all jackknife samples.

We used the bias corrected confidence interval to test the null hypothesis that the ECI value is not different from zero, in other words, there is no equivalent or inverse change between the effect sizes of the attributes of the two studies. We reject the null hypothesis when the confidence interval of the ECI values does not include zero.

#### 2.2.2 P-value (approximate)

According to Asparouhov and Muth´en (Tihomir Asparouhov aBMe, 2021), assuming that $\hat{\text{a}}=0$ , we have

$${\text{a}}_{1}\;=\;\text{Φ}\left({\hat{\text{z}}}_{0}+{\hat{\text{z}}}_{0}+{\text{z}}^{\left(\text{α}/2\right)}\right)\;=\;\text{Φ}\left(2{\hat{\text{z}}}_{0}+{\text{z}}^{\left(\text{α}/2\right)}\right)$$

$${\text{a}}_{2}\;=\text{Φ}\left({\hat{\text{z}}}_{0}+{\hat{\text{z}}}_{0}+{\text{z}}^{\left(1-\text{α}/2\right)}\right)\;=\;\text{Φ}\left(2{\hat{\text{z}}}_{0}+{\text{z}}^{\left(1-\text{α}/2\right)}\right)$$

When we concentrate only on positive ECI values, we would reject the null hypothesis of non-equivalence when ${\hat{\text{β}}}_{\text{L}}^{\text{*}}>0$ or L > M where

$$M=\overset{B}{\underset{b=1}{\sum }}I_{\left[{\hat{\text{β}}}^{*}<0\right]}$$

In the special case of *p*-value = 0.05 (L = M) we have

$$\begin{array}{l} 0.5=2\cdot 0.025=2\cdot \text{Φ}\left(-1.96\right)\\ =2\cdot \text{Φ}\left(-2{\hat{\text{z}}}_{0}+2{\hat{\text{z}}}_{0}-1.96\right)\\ =2\cdot \text{Φ}\left(-2{\hat{\text{z}}}_{0}+\Phi^{-1}\left(\Phi \left(2{\hat{\text{z}}}_{0}-1.96\right)\right)\right)\\ =2\cdot \text{Φ}\left(-2{\hat{\text{z}}}_{0}+\Phi^{-1}\left({\text{a}}_{1}\cdot B/B\right)\right)\\ =2\cdot \text{Φ}\left(-2{\hat{\text{z}}}_{0}+\Phi^{-1}\left(L/B\right)\right) \end{array}$$

Therefore, the two-sided *p*-value can be computed, approximately, by

$$p-value\;=\;2\cdot \text{Φ}\left(-2{\hat{\text{z}}}_{0}+\Phi^{-1}\left(M/B\right)\right)$$

where M is the number of ECI values, which are smaller than zero. In case of the ECI value being smaller than zero we compute

$$p-value\;=\;1-2\cdot \text{Φ}\left(-2{\hat{\text{z}}}_{0}+\Phi^{-1}\left(M/B\right)\right)$$

### 2.3 Two one-sided t-tests

What follows is a brief description of how the TOST method was applied to these data. Let Δ represent some fold change which is considered to be unimportant. To test for equivalent change, we use a null hypothesis of non-equivalence vs. an alternative hypothesis of equivalence or inverse relationship. In particular, we must perform two hypothesis tests, namely:

$${\text{H}}_{01}:{\text{β}}_{\text{i}1}-{\text{β}}_{\text{i}2}\geq \Delta \;\mathrm{vs}\;{\text{H}}_{\text{a}1}:{\text{β}}_{\text{i}1}-{\text{β}}_{\text{i}2}<\Delta$$

and,

$${\text{H}}_{02}:{\text{β}}_{\text{i}1}-{\text{β}}_{\text{i}2}\leq -\Delta \;\mathrm{vs}\;{\text{H}}_{\text{a}2}:{\text{β}}_{\text{i}1}-{\text{β}}_{\text{i}2}>-\Delta$$

where ${\text{β}}_{\text{ik}}$ is the effect size of gene i of study k . The *t*-test statistics for the two tests are:

$${\text{t}}_{\text{i}}^{\text{*}1}=\frac{{\hat{\text{β}}}_{\text{i}1}-{\hat{\text{β}}}_{\text{i}2}-\Delta }{{\text{s}}_{\text{i}}}$$

and,

$${\text{t}}_{\text{i}}^{\text{*}2}=\frac{{\hat{\text{β}}}_{\text{i}1}-{\hat{\text{β}}}_{\text{i}2}+\Delta }{{\text{s}}_{\text{i}}}$$

To accommodate for unequal variances we define ${\text{s}}_{\text{i}}$ according to Welch’s *t*-test, where ${\text{s}}_{\text{i}}=\sqrt{\frac{sd_{1}^{2}}{N_{1}}+\frac{sd_{2}^{2}}{N_{2}}}$ , $sd_{k}$ is the standard deviation of ${\hat{\text{β}}}_{\text{i}k}$ , and $N_{k}$ is the size of study k . To perform the *t*-test we used Satterthwaite formula for degrees of freedom:

$$df=\frac{{\left(\frac{sd_{1}^{2}}{N_{1}}+\frac{sd_{2}^{2}}{N_{2}}\right)}^{2}}{\frac{{\left(\frac{sd_{1}^{2}}{N_{1}}\right)}^{2}}{N_{1}-1}+\frac{{\left(\frac{sd_{2}^{2}}{N_{2}}\right)}^{2}}{N_{2}-1}}$$

Only when the *p*-value for test one and the *p*-value for test two are smaller than the significance level of α=0.05 , is a gene considered to be equivalently changed. Likewise, to test for inverse change, we use the same test statistics except that we multiply ${\hat{\text{β}}}_{\text{i}1}$ by −1.

### 2.4 Multiple testing

We performed a test of equivalence for each gene. To adjust the *p*-value for the multiple testing we used the adjusted false discovery rate (FDR) approach by Benjamini and Hochberg (1995). Here, the FDR correction $q_{i}=p_{i}N/i$ , where $p_{i}$ is the *i*th *p*-value in a sorted list of ascending *p*-values and N is the total number of *p*-values, is the ratio of expected false positives and the total number of accepted positives. To adjust for the non-monotony of the $q_{i}$ value we replace the $q_{i}$ value with the lowest $q_{i}$ value among all $q_{f}$ , where f≥i (Yekutieli and Benjamini, 1999).

### 2.5 Simulation

We conducted a simulation study to compare the two different tests for equivalent change. This simulation study consisted of two simulated studies of differential gene expression, for which we aimed to test equivalent change of gene expression. The two simulated studies, each consisting of 1,000 genes, are constructed so that 30% of the differentially expressed genes of Simulated Study 1 are equivalently changed between the two studies. The simulation process has two levels. First, we simulated descriptive features of each simulated study (mean and standard deviation), which we used in the second level to draw random gene expression values for each sample. We then used the simulated studies to performed differential gene expression and the equivalence testing as described above. In the following we will explain how each study is set up step by step.

#### 2.5.1 Simulated study 1

The first stage is to simulate a study with two groups. The group is denoted by k , with controls as k=0 and cases or treatment as k=1 . The simulation derives certain values from a reference study of pancreatic cancer (see section Biological Data) with case control data, including samples that were either tumor or tumor adjacent normal tissue (GSE16515), as described below.

a. The mean expression for gene g in the simulated control group (k=0) is drawn from a gamma distribution with scale and shape parameters extracted from the reference dataset using the function egamma () of the R package EnvStats (Millard, 2013):

$$m_{g}∼\text{Γ}\left(\alpha =8.24,\;\beta =0.66\right)$$

b. For each gene g from the reference dataset we obtained the mean difference in expression, denoted by $\delta_{g}$ .
c. Genes with $-1\geq \delta_{g}\leq 1$ are removed. For this simulation we are only interested in genes with a difference between groups.
  d. From the filtered down gene set we obtained a new gene set by sampling with replacement, where each gene g has:
  i. The standard deviation for gene g in group k of the reference dataset, denoted by $s_{gk}$

  ii. The mean difference $\delta_{g}$

e. The genes were divided into three subgroups: equivalently changed genes (f = 1), non-equivalently changed genes 1 (f = 2), and non-equivalently changed genes 2 (f = 3). The non-equivalently changed genes were divided into two subgroups to have the differential expression of those genes be balanced between the two studies: One half of the genes is differentially expressed in one study while non-differentially expressed in the other study and vice versa.
f. The expression value for gene g , observation i , group k is denoted $x_{gik}$ and is drawn from a truncated normal distribution:

$$x_{gik}∼N\left(\gamma_{g},s_{gk}\right);0<x_{gik}$$

• With

$$\gamma_{g}=\{\begin{array}{cc} m_{g}+k\delta_{g} & if\;f=1\\ m_{g} & if\;f=2\\ m_{g}+k\delta_{g} & if\;f=3 \end{array}$$

g. With the simulated control group and treatment group, we calculated the differential expression for each gene as described in section “Differential Gene Expression”.

#### 2.5.2 Simulated study 2

The next stage is to simulate gene expression from a second, similar study. At this point, we will determine genes which are equivalently changed across the two studies. We will choose 30% of the differentially expressed genes (abs (log2FC) > 1 and p-value < 0.05) from Simulated Study 1 to be equivalently changed.

a. The mean expression for a gene in the simulated control group is equal to the mean expression in Simulated Study 1.
b. Equivalently changed genes are further simulated to not always be perfectly equivalently changed (even on average). This is done by having a modifier for the change in gene g , denoted $\theta_{g}$ and is in [1,2.5].
c. For Simulated Study 2, the expression value for gene g , observation j , group k is then drawn from a truncated normal distribution:

$$y_{gik}∼N\left(\gamma_{g},s_{gk}\right);0<y_{gik}\;$$

With

$$\gamma_{g}=\{\begin{array}{cc} m_{g}+k\theta_{g}\delta_{g} & if\;f=1\\ m_{g}+k\delta_{g} & if\;f=2\\ m_{g} & if\;f=3 \end{array}$$

We used the pairing of $s_{gk}$ and $\delta_{g}$ to be able to simulate the fact that differentially expressed genes with low difference in means between the treatment groups often have small standard deviations.

**FIGURE 2:**
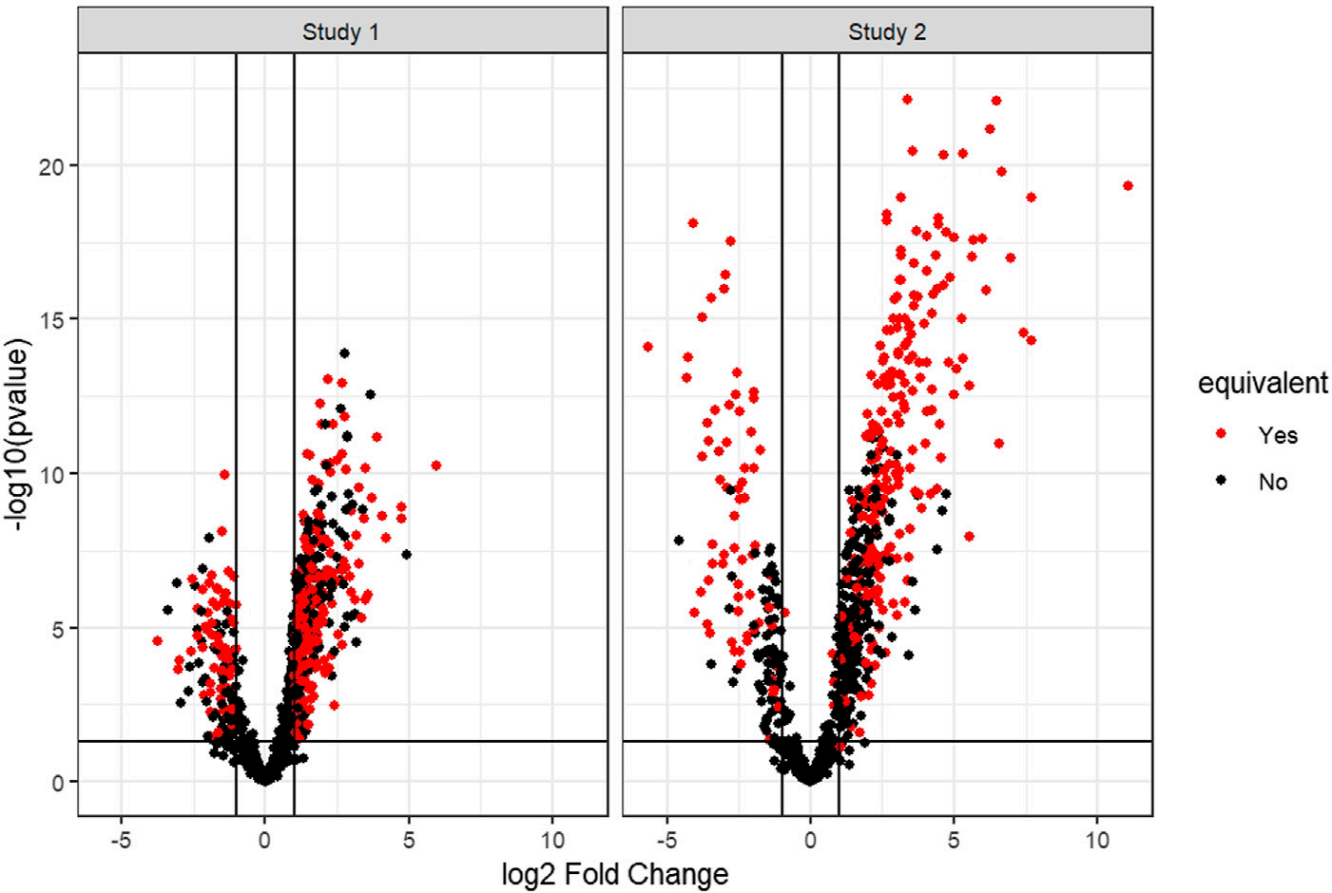
Example volcano plots of the two simulated gene expression studies of one simulation iteration. Marked red are all genes set to be equivalent between the two studies. Here, the differential expression of the genes to be set equivalently changed between the two simulated studies must pass a threshold (abs (log2FC) > 1 and *p*-value < 0.05) in Simulated Study 1 but the differential expression of the same genes is altered in Simulated Study 2 to mirror equivalent change and thus can fail the threshold for differential gene expression.

Figure 2 shows the distribution of differential gene expression of the two simulated studies for one iteration of the simulation. The degree of equivalence of change in gene expression between the two studies for one iteration of the simulation is visualized in Supplemental Figure S1.

### 2.6 Performance measures

To assess the performance of each test we used sensitivity, specificity, balanced accuracy and F1 score, which is the harmonic mean of precision and sensitivity.

### 2.7 Biological data

The biological data analysis is based on publicly available pancreatic cancer microarray expression data, Alzheimer’s disease microarray expression data, and smoking RNAseq data obtained from the NCBI-GEO database (Edgar et al., 2002; Barrett et al., 2013) (Gene Expression Omnibus (GEO), RRID:SCR_005012). Given that these datasets are drawn from similar populations, we expect to see equivalently changed genes among them.

For pancreatic cancer, one dataset was comprised of samples of peripheral blood mononuclear cells (PBMC) (accession number GSE74629). The other two datasets are comprised of samples of tumor tissue (tumor tissue data set 1: GSE16515, tumor tissue data set 2: GSE22780) (Pei et al., 2009; Ellsworth et al., 2013; Li et al., 2016) (Table 1). The control data for the PBMC study were from gender, age, and habit matched healthy participants and the control data for the two disease tissue studies are from adjacent normal tissue of the cancer patients. After reading in the raw files, the count data were background corrected, quantile normalized, and the expression values were calculated using the function rma () from the package affy (Gautier et al., 2004) for Affymetrix microarray data, or the function neqc () from the package limma (Ritchie et al., 2015) for Illumina microarray data in the R statistical environment.

**TABLE 1:** Specifics for the different pancreatic cancer studies used in this project.

Tissue type	Accession #	# Tumor	# Control
Disease	GSE16515	36	16
Disease	GSE22780	8	8
PBMC	GSE74629	36	14

For Alzheimer’s disease, the studies GSE1297 and GSE29378 were compared. In GSE1297 only severe cases vs. control were considered since GSE29378 only included advanced Alzheimer’s disease cases. In GSE29378 we only used the CA1 regions, in order to match what was used for GSE1297. GSE1297 was background subtracted and normalized using the rma () function from the affy package and GSE29378 was background corrected and normalized using neqc () function from the limma package.

For the smoking data set, the study GSE169757 was used where smokers were compared to control and vapers. We used the function cpm () from the edgeR packet (Robinson et al., 2010; McCarthy et al., 2012) to filter expressed genes by a threshold of 0.3, which roughly corresponds to a count of 10. To normalize the data, we used the function calcNormFactors () from the edgeR package and voom () from the limma package.

### 2.8 Differential gene expression

We used the package limma to perform differential gene expression analysis. The model was fitted by using the functions lmfit () and eBayes (), which uses moderated t-statistics for ranking the genes. The standard deviation (sd) for gene g was be extracted from the output by multiplying the square root of the unscaled covariance $\vartheta_{\text{gj}}$ and the posterior residual variance ${\tilde{\text{s}}}_{\text{g}}^{2}$ , $\text{sd}=\sqrt{\vartheta_{\text{gj}}}\cdot {\tilde{\text{s}}}_{\text{g}}$ as recommended by Smyth (2004).

### 2.9 Disease enrichment analysis

To investigate the association of equivalently changed genes with disease types we used the R package DOSE (Yu et al., 2015), which calculates a *p*-value using the hypergeometric distribution to determine whether the number of genes associated with a disease is larger than expected. We used the function enrichDO (), which supports Disease Ontology (DO) data (Schriml et al., 2012), and enrichDGN, which supports DisGeNET (Pinero et al., 2015) (DGN), with a minimal size of genes annotated by NCG category for testing of 5 and a q-value cut off of 0.05.

## 3 Results

### 3.1 Simulation

**FIGURE 3:**
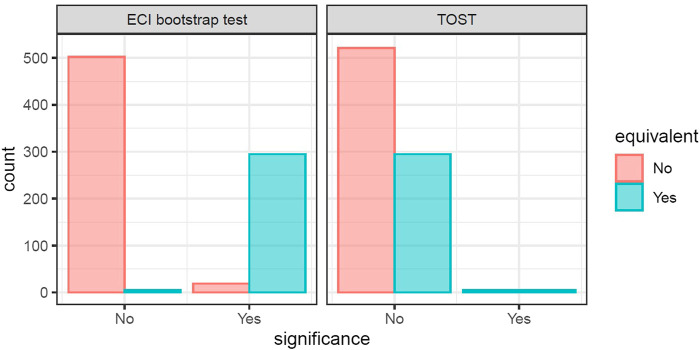
Example plots of decisions based on the q-value by the two tests of one simulation iteration. Left is the significance decision of the ECI bootstrap test, right is the significance decision of the TOST.

Simulation 1 was comprised of Simulated Study 1 and Simulated Study 2. Each simulated study contained a simulated treatment and control. The two simulated studies were created such that 30% of the genes were equivalently changed with varying degrees of equivalence. Figure 3 and Table 2 show the decision of significance of the two tests (ECI bootstrap test and TOST) for one single iteration of the simulation. As can be observed, for a sample size of the comparator groups (e.g., case vs. control) of 20 people in each of the control group and case group, the ECI bootstrap test is able to identify the majority of equivalently changed genes (in this example sensitivity = 0.987) but also falsely identifies not equivalently changed genes (specificity <1). TOST on the other hand, does not misclassify any non-equivalently changed genes but identifies only 5 equivalently changed genes.

**TABLE 2:** Performance measurements for one single simulation with group size 20. Decisions of equivalence are made using the q-value of 0.05 for ECI bootstrap test and TOST. The TOST is too conservative to identify any equivalently changed genes, while the ECI bootstrap test shows good performance.

Performance	ECI bootstrap test	TOST
Sensitivity	0.987	0
Specificity	0.951	1
Balanced accuracy	0.969	0.5
F1-score	0.953	0

**FIGURE 4:**
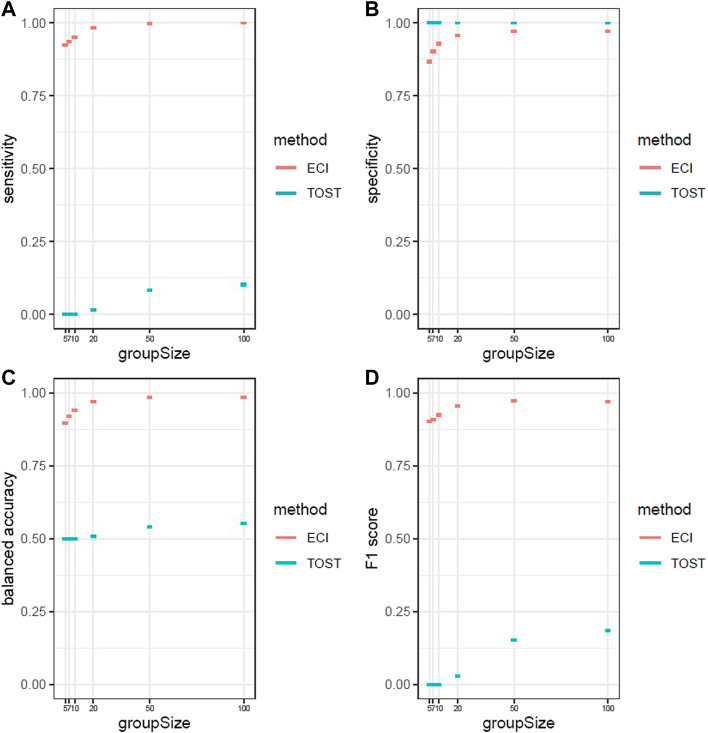
Performance metrics with 95% confidence intervals of 1,000 iterations for ECI bootstrap test and two one-sided *t*-test **(A)** Sensitivity **(B)** specificity) **(C)** balanced accuracy **(D)** F1 score. The ECI bootstrap test shows increasing performance with increasing group size and has overall a good performance. The TOST shows only in specificity a better performance than the ECI bootstrap test and shows no performance for all the other measurements.

We repeated the previously described simulation 1,000 times and calculated the average of sensitivity, specificity, balanced accuracy, and F1 score for different sample sizes of the comparator groups, namely 5, 7, 10, 20, 50, and 100 (Figure 4). As can be seen, the sensitivity and F1 score for TOST are less than 0.2 and balanced accuracy is close to 0.5 irrespective of group size, and the specificity is close to 1 for all group sizes. The ECI bootstrap test shows that all performance metrics are dependent on the group size (as expected). Nevertheless, the performance of the ECI bootstrap test is overall very good. For a group size of 10, the balanced accuracy averages 0.940 and the F1 score averages 0.925 (Supplemental Table S1).

### 3.2 Biological data

In the following, the naïve approach is defined as declaring a gene equivalently changed when it is differentially expressed in the same direction in both studies. For the ECI bootstrap test and TOST we report a gene as significant when it differentially expressed in at least one of the two studies and has a q-value less than 0.05.

#### 3.2.1 Pancreatic cancer

We used three publicly available data sets that were created to study pancreatic cancer. Two of the datasets contained gene expression measurements in pancreatic tumor tissue with adjacent normal tissue as control and the third data set contained gene expression measurements from peripheral blood mononuclear cells (PBMCs) with gene expression data from gender, age, and habits matched healthy patients as controls (hereafter referred to as the PBMC data set).

We performed bootstrap ECI test, TOST, and naïve approach on the biological data. Using TOST, none of the genes were significantly equivalently changed between the two tumor tissue data sets. When using the naïve approach, 150 genes were identified as equivalent. While using bootstrap ECI, the comparison of the two tumor tissue data sets led to the identification of 251 genes which were equivalently changed. From the 150 genes defined equivalent by the naïve approach, 127 were found equivalently changed using ECI bootstrap test (Supplemental Table S2). Significant genes identified by the ECI bootstrap test are enriched with annotations for pancreatic cancer (p-adj = 5.5e-04), pancreatic carcinoma (p-adj = 3.4e-03), and pancreatic adenocarcinoma (p-adj = 7.6e-03) when using the DO database (Supplemental Table S7). When we use the DGN database, multiple disease descriptions were related to cancer such as adenocarcinoma of pancreas (p-adj = 2.0e-04), or gastric adenocarcinoma (p-adj = 6.4e-03) (Supplemental Table S8). The top 10 equivalently changed genes include some with known or suspected tumor relevance such as CYP3A5 (Noll et al., 2016), STEAP1 (Gomes et al., 2012), and PROX1 (Saukkonen et al., 2016), which are being investigated as drug targets for pancreatic cancer, GMNN (Kim et al., 2012; Kushwaha et al., 2016), which is associated with cancer pathophysiology and development, and SORBS2 (Alsafadi et al., 2011; Zhao et al., 2018; Lv et al., 2020; Lv et al., 2021), which is known to be associated with metastatic relapse.

Using TOST we identified two equivalently changed genes (SPICE1 and MCEMP1) and one inverse changed gene (STAMBPL1) by comparing tumor tissue data set 1 with the PBMC data set. When using the naïve approach, 14 genes were found equivalently changed. When using the ECI bootstrap test, we identified 181 equivalently changed genes and 94 inversely changed genes and all genes equivalently changed using the naïve approach were also identified as equivalently changed using the ECI bootstrap test (Supplemental Table S3). The inversely changed genes were not found to be associated with diseases listed in either the DO database or the DGN database. Significant equivalently changed genes identified by the ECI bootstrap test are enriched with annotations for pancreatic cancer (p-adj = 3.2e-03), pancreatic carcinoma (p-adj = 2.1e-02), and pancreatic ductal adenocarcinoma (p-adj = 1.3e-02) when using the DO database (Supplemental Table S9). When using the DGN database, multiple disease descriptions related to pancreatic cancer were identified such as pancreatic ductal adenocarcinoma (*p*-value = 1.3e-05), and adenocarcinoma of pancreas (*p*-value = 2.3e-03) (Supplemental Table S10).

The comparison of tumor tissue data set 2 with the data set from PBMC let to no equivalently changed genes being identified when using TOST, two equivalently changed genes identified when using naïve approach, and we identified nine equivalently changed genes and eight inversely changed genes when using the ECI bootstrap test. The two genes identified as equivalently changed when using the naïve approach were also significantly equivalently changed using the ECI bootstrap test (Supplemental Table S4). Using the DO database as well as the DGN database resulted in no disease descriptions found to be associated with the equivalently changed genes, likely due to the small number identified.

#### 3.2.2 Alzheimer’s disease

We compared two publicly available data sets for Alzheimer’s disease, where 147 genes were differentially expressed in at least one of the two studies. We found no genes to be equivalently changed using TOST and three genes identified as equivalently changed using the naïve approach. Using the ECI bootstrap test we found 20 genes to be equivalently changed, where two were differentially expressed in both studies (ANO3 and SST), and 1 gene to be inverse changed (Supplemental Table S5). The equivalently changed genes are enriched with an annotation for Non-Functioning Pituitary Gland Neoplasm (p.adj = 0.039) when using the DGN database (Supplemental Table S11), where down-regulation of the Pituitary Gland is associated with Alzheimer’s disease (Ishii and Iadecola, 2015).

The top equivalently changed genes identified by the ECI bootstrap test are: SST, FXYD7, PCP4, RASL12, SLC14A1, INA, SERPINA3, ANO3, APLNR, AEBP1. SST is directly linked to AD (Solarski et al., 2018). FXYD7, APLNR, INA, AEBP1 and SERPINA3 were found to be differentially expressed in a meta-analysis of AD data sets (Su et al., 2019). PCP4 was found to be dysregulated in the forebrain of mice with AD (Renelt et al., 2014). SLC14A1 is one of significantly altered transcripts in APPswe/PS1dE9 transgenic mice during the development of beta amyloid protein (Aβ) pathology (Wirz et al., 2013). Finally, ANO3 is significantly differentially expressed in relation to Alzheimer’s disease (Vastrad and Vastrad, 2021).

#### 3.2.3 Smoking vs. vaping

We used one publicly available data set of a study of three groups: smoking, vaping, control. We compared the control group against both smoking and vaping for differential gene expression and finally compared the two sub-studies (smoking vs. control and vaping vs. control) using the TOST and ECI bootstrap test. No genes were found to be equivalently changed using the TOST and 382 genes were found to be equivalently changed using the naïve approach. Using the ECI bootstrap test we found 1,090 genes to be equivalently changed and 0 to be inversely changed (Supplemental Table S6). Out the 1,090 genes, 380 genes were differentially expressed in both studies, 404 were protein coding genes and 348 could be used for disease enrichment analysis because they had an entrez id. The disease enrichment analysis delivered 0 associated diseases for both the DO database and the DGN database.

The top 10 equivalently changed genes are: TERB2, TM4SF1, PHKA1, GLYATL1, AC073610.2, PF4, HECW1, CALD1, APOBEC4, NPHP3-ACAD11. TERB2 is associated with Spermatogenic Failure 59 (GeneCards, 2022). (Dai et al., 2015) found that smoking is associated with impaired spermatogenesis. (Fu et al., 2020). found TM4SF1 to be associated with smoking. PHKA1, an inflammatory-associated transcript, is significantly altered with prenatal nicotine exposure (Zhou et al., 2021). GLYATL1 is associated with HCC (Guan et al., 2020). PF4 is found to be significantly higher expressed when smoking (Zevin et al., 2001). CALD1 is associated with *in utero* tobacco smoke exposure (Breton et al., 2014). And two SNPs nearest to APOBEC4 were associated with fluorescent oxidation products (FIOPs) accounting for tobacco smoking status in adults (Orsi et al., 2022).

## 4 Discussion

In this work, we compared two options to test for equivalently changed genes between two studies, namely the proposed ECI bootstrap test and TOST. We were able to show that the ECI bootstrap performs well in identifying equivalently changed genes with respect to balanced accuracy and F1, which were on average both over 0.9, even for the smallest analyzed group size of ten, while maintaining a high specificity. Furthermore, we found that TOST greatly underperformed the ECI bootstrap test with balanced accuracy close to 0.5 and F1 score close to 0.

By using a threshold of 0.05 for the q-value, for the ECI bootstrap test, we were able to identify many equivalently changed genes between two pancreatic cancer tumor tissue studies where most of the genes were related to cancer progression. When comparing two Alzheimer’s data sets, the majority of the equivalently changed genes were associated with Alzheimer’s disease. The lack of significant enrichment for Alzheimer’s disease itself could simply be due to non-comprehensive annotation in the databases used. Additionally, when we were comparing smoking to vaping most of the top equivalently changed genes were related to smoking, though the enrichment analysis delivered no associated disease. This is not surprising, given that vaping is not necessarily expected to lead to similar health outcomes as smoking. Nevertheless, the results show that the ECI bootstrap test met our expectation of identifying equivalently changed genes in studies of the same disease type or exposure and, furthermore, is able to identify equivalently changed genes which are functionally related to the disease type or exposure and could lead to more reproducible or robust results. Additionally, we were able to show that several genes in non-cancer tissue (peripheral blood) of patients of pancreatic cancer showed equivalently changed behavior to genes of tumor tissue of the same disease which implies the systemic impact of cancer. Those results may open the way for identifying reliable blood markers for cancer, and for new investigative approaches into the field of systemic changes of gene expression of cancer patients that may be addressed in future studies. It must be mentioned that the difference in the number of significant genes could be due to the group sizes of the data sets.

When comparing the ECI bootstrap test to the naïve approach we found several genes which were declared equivalently changed using the naïve approach to be not significant using the ECI bootstrap test. In particular, 23 genes out of 150 genes differentially expressed in both pancreatic tumor tissue studies were not significantly equivalently changed when using the ECI bootstrap test. This shows that simply identifying genes as equivalently changed because they are differentially expressed in the same direction might lead to false conclusions. On the other hand, in the comparison of vaping vs. smoking only 2 out of 382 genes differentially expressed in both studies were not significantly equivalently changed using the ECI bootstrap test. One reason for that could be an inherent bias due to using the same control as comparison. Additionally, the ECI bootstrap test is more powerful because it can identify statistically equivalently changed genes even when they are not statistically significantly changed in one of the studies. Furthermore, although we have demonstrated the situation in which we test for any degree of equivalent change, it is just as simple to test for the desired level of equivalent change, which the naïve method cannot do. Overall, the ECI bootstrap test gives more reliable results when identifying equivalently changed genes in comparison to the naïve approach.

Limitations of this study include the differing nature of cases and controls in some datasets, the different group sizes, and the lack of a ground truth in biological data. In future work, the plan is to create a framework for power calculation to address the influence of different group sizes on the ECI bootstrap test performance.

## 5 Conclusion

In this study we demonstrated the use of the ECI bootstrap test in a setting of differentially expressed genes to provide researchers with a statistical approach to identify genes which are similarly influenced by a specific treatment or exposure. Furthermore, statistically identified equivalently changed genes reduces the cost for validating those genes and offers the option of identifying new possible treatment targets.

In addition, it is also possible, due to the non-parametric nature of the bootstrap test and the lack of assumptions on the ECI value, to adapt the ECI bootstrap test to other options such as methylation data or other types of ‘omics data. In future studies we want to investigate the effectiveness of bootstrap ECI on other types of data sets.

Furthermore, we have created an R package for the ECI bootstrap test which can be obtained from the github repository at https://github.com/Hecate08/ECIbootstrap.

## Data Availability

The datasets analyzed in this study can be found in the NCBI repository https://www.ncbi.nlm.nih.gov/geo/query/acc.cgi?acc=GSE16515, https://www.ncbi.nlm.nih.gov/geo/query/acc.cgi?acc=GSE22780, and https://www.ncbi.nlm.nih.gov/geo/query/acc.cgi?acc=GSE74629.

## Abbreviations

DO, Disease Ontology; DGN, DisGeNET; ECI, Equivalent Change Index; PBMC, peripheral blood mononuclear cells; TOST, Two One-Sided t-Tests.
