# Supplementary material for: Assessing equivalent and inverse change in genes between diverse experiments
Source: Front Bioinform. 2022 Sep 21;2:893032. doi: 10.3389/fbinf.2022.893032 (PMC9580844; doi:10.3389/fbinf.2022.893032)
Supplement: Supplementary file 11 [file DataSheet1.DOCX]

Supplemental data


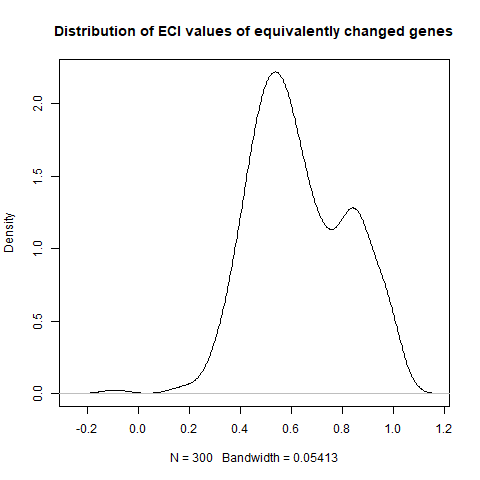


F1: Distribution of ECI values of genes which are equivalently changed between between the two simulated studies.

T1: Performance metrics of the different tests for different group sizes.

|  | ECI bootstrap | | | | TOST | | | |
| --- | --- | --- | --- | --- | --- | --- | --- | --- |
| groupSize | Sensitivity | Specificity | Balanced accuracy | F1 score | Sensitivity | Specificity | Balanced accuracy | F1 score |
| 5 | 0.925 | 0.868 | 0.896 | 0.902 | 0.000 | 1.000 | 0.500 | 0.000 |
| 7 | 0.936 | 0.902 | 0.919 | 0.909 | 0.000 | 1.000 | 0.500 | 0.000 |
| 10 | 0.950 | 0.929 | 0.940 | 0.925 | 0.000 | 1.000 | 0.500 | 0.000 |
| 20 | 0.983 | 0.958 | 0.971 | 0.957 | 0.015 | 1.000 | 0.507 | 0.029 |
| 50 | 0.997 | 0.971 | 0.984 | 0.972 | 0.083 | 1.000 | 0.541 | 0.152 |
| 100 | 1.000 | 0.971 | 0.985 | 0.971 | 0.103 | 1.000 | 0.551 | 0.186 |
